# Antimicrobial Activities Evaluation and Phytochemical Screening of Some Selected Plant Materials Used in Traditional Medicine

**DOI:** 10.3390/molecules28010244

**Published:** 2022-12-28

**Authors:** Kinga Stuper-Szablewska, Tomasz Szablewski, Anna Przybylska-Balcerek, Lidia Szwajkowska-Michałek, Michał Krzyżaniak, Dariusz Świerk, Renata Cegielska-Radziejewska, Zbigniew Krejpcio

**Affiliations:** 1 Department of Chemistry, Poznań University of Life Sciences, 60-637 Poznan, Poland; 2 Department of Food Quality and Safety Management, Poznań University of Life Sciences, 60-637 Poznan, Poland; 3 Department of Landscape Architecture, Poznań University of Life Sciences, 60-637 Poznan, Poland; 4 Department of Human Nutrition and Dietetic, Poznań University of Life Sciences, 60-637 Poznan, Poland

**Keywords:** antimicrobial properties, bioactive compounds, natural antioxidants, bitter melon, elderberry, wild rose, mountain ash, guelder rose, sea buckthorn

## Abstract

Plant extracts are a source of valuable ingredients that can be used in many industries. This paper presents research on the content of selected bioactive compounds in extracts obtained from various plant materials. Raw materials have a documented use in traditional medicine not only in Poland. The tested plants were: bitter melon (fruit), elderberry (flowers, fruit, leaves), wild rose (fruit, flesh, seeds), mountain ash (fruit), guelder rose (fruit), and sea buckthorn (fruit, leaves, pomace). The main goal of these tests is to indicate the potential raw materials that may constitute an alternative source of bioactive compounds with antimicrobial activity. The plant material was tested for the content of bioactive antioxidant compounds and possible antimicrobial activity. The content of polyphenols (phenolic acids and flavonoids) was analyzed using UPLC/PDA, sterols, organic acids, and other bioactive compounds. The minimum inhibitory concentration (MIC) was determined. The total free phenolic acids (TPC) and total free flavonoids (TFC) of all plant raw materials was varied and ranged from 0.21 (mg RUTE/1 g of extract) to 38.30 mg RUTE/1 g of extract) for TFC. The concentration of sterols was, on average, about 10 mg/1 g of extract. The value of approx. 20 mg/1 g of the extract was recorded for bitter melon and beach rose. The content of organic acids was about 1.5 mg/1 g of the extract to even 13 mg/1 g of the extract for sea buckthorn berries. The most sensitive to the extracts’ activity were the following bacteria: *M. luteus*, *P. mirabilis*, *P. fragii*, *S. enteritidis,* and *E. coli*. The tested plant materials can be used in various industries as a source of bioactive compounds of an antibacterial nature.

## 1. Introduction

There are between 250,000 and 500,000 plant species on Earth, of which humans use a relatively small percentage (1% to 10%). Compounds synthesized by plants perform various functions in human metabolism. For example, therapeutic or toxic secondary metabolites (excluding nutritional functions), found in plants or their extracts in small amounts, are bioactive compounds [1]. Due to the diverse chemical structure, the metabolic pathways in which they arise or the biological functions they perform can be classified according to various criteria. One of the most frequently cited is the division proposed in 2000 by Croteau [2]. He divided them into three categories: terpenes and terpenoids (about 25,000 different hydrocarbons and their derivatives), alkaloids (about 12,000 basic compounds, generally nitrogen-containing heterocycles), and phenolic compounds (about 8000 arenes, containing a hydroxyl-OH group bonded directly to the aromatic ring) [3]. These bioactive ingredients are obtained from plant extracts using various extraction techniques [4,5,6].

Plant extracts are a source of valuable ingredients that can be used in many industries [7,8]. In recent years, much attention has been paid to products based on natural substances and their use possibility in various industries, including cosmetics and pharmacy. Therefore, new plant materials are sought to be a source of bioactive compounds with antioxidant and antimicrobial properties.

This paper presents research on the content of selected bioactive compounds in extracts obtained from various plant materials. Raw materials rich in polyphenols, which have a documented use in traditional medicine not only in Poland, were selected. The tested plants and materials were: bitter melon (fruit), elderberry (flowers, fruit, leaves), wild rose (fruit, flesh, seeds), mountain ash (fruit), guelder rose (fruit), and sea buckthorn (fruit, leaves, pomace). An additional exciting research aspect was the analysis of extracts obtained from various parts of the fruit: seeds, pomace, flowers, and leaves. The main goal of these studies is the indication of potential raw materials that may constitute an alternative source of bioactive compounds with antimicrobial activity. Research on these raw materials has not been conducted so far, so it is an unexplored area.

The first plant proposed is bitter melon (*Momordica charantia* L.). It has a hypoglycemic and antimicrobial effect, affects the immune system functioning, and inhibits specific cancers’ development [9,10].

Another raw material is beach rose (*Rosa rugosa* Thunb.) fruit, seeds, and pomace. Of all the Central European climate zone plant raw materials, beach rose fruits contain the most vitamin C. Tocopherols, carotenoids, and phenolic acids, as well as complementing the vitamin composition. Thanks to this composition, *Rosa rugosa* has antifungal and antiviral properties [11,12].

The next group of samples is elderberry (*Sambucus nigra* L.) flowers and leaves used in traditional medicine to treat inflammation, skin diseases, diuretics, colds, fever, and other respiratory disorders.

Another plant with confirmed antioxidant properties is mountain ash (*Sorbus aucuparia* L.), commonly known as rowan. In the course of the conducted research, it was found that its fruits are a potential source of substances that can be used to treat cancer and diseases of the digestive, neurological, and cardiovascular systems. Rowan fruits contain organic acids, phenolic acids, flavonoids, anthocyanins, tannins, and many other compounds.

Guelder rose (*Viburnum opulus* L.) is a plant rich in phenolic compounds. Chlorogenic acid is mainly responsible for its antioxidant effect. It also has anti-inflammatory properties [13].

Sea buckthorn (*Hippophaë rhamnoides* L.) belongs to the *Elaeagnaceae* family. It is characterized by a rich content of biologically active compounds and shows a wide range of activities. These compounds are micronutrients and vitamins (mainly A, C, and E), lipids, carotenoids, amino acids, unsaturated fatty acids, and phenols [14,15,16,17].

The content of bioactive compounds in these raw materials has so far been analyzed from methanolic extracts and mainly concerned the content of free bioactive compounds. Thanks to the proprietary method described by Przybylska-Balcerek et al. [18] we can quantify the most important bioactive compounds, including those related. As for the antimicrobial activity, this paper will present the research results together with the composition of the extract.

Flavonoids are among the important antioxidant and biologically active compounds. They are a group of chemical compounds that act as dyes in the plant. They give plants intense colors from yellow to orange. They are accumulated in the surface layers of the plant, and thus protect them from UV radiation. They also act as “plant protectors”—they repel insects and fungi. Particularly rich in flavonoids is, for example, the flower of the tricolor violet (*Viola tricolor*) or elderberry (*Sambucus nigra*). One of the representatives of flavone compounds—rutin, can also be found in buckwheat (*Fagopyrum*). It has been used in medicine since the 19th century. It strengthens blood vessels, has an antioxidant effect, increases immunity, and accelerates metabolism. Other medicinal plants due to the content of flavonoids are parsley (*Petroselinum crispum*) or horsetail (*Equisetum arvense*).

The second important group are phenolic acids, which are compounds with hydroxyl and carboxylic functional groups. Among the phenolic acids, the most important have derivatives of benzoic acid, e.g., caffeic, ferulic, and sinapic acids, and cinnamic acid derivatives, e.g., acids gallic, protocatechol, and syringic. Phenolic acids are a group of secondary metabolites widely distributed in the plant world, which show a positive impact on human health. They are considered enriching ingredients and precursors of flavoring substances, most of all for bioactive compounds. They are characterized by a wide spectrum of pharmacological activity and are primarily responsible for the removal of free radicals, chelation of metal ions, and changes in enzyme activity. They exhibit antibacterial activity and have anti-allergic, anti-viral, and odor-smelling properties.

The study aimed to determine the MIC (minimum inhibitory concentration) of extracts obtained from various medical plants’ raw materials, analyze their composition, and determine the relationship between the content of individual bioactive compounds and the antimicrobial activity of the studied extracts.

## 2. Results and Discussion

The research presented in this paper was carried out in two ways. First, microbiological analyses were used to determine the MICs against selected microorganisms. The chemical analyses were performed to characterize the extracts used in microbial tests.

**Figure 1:**
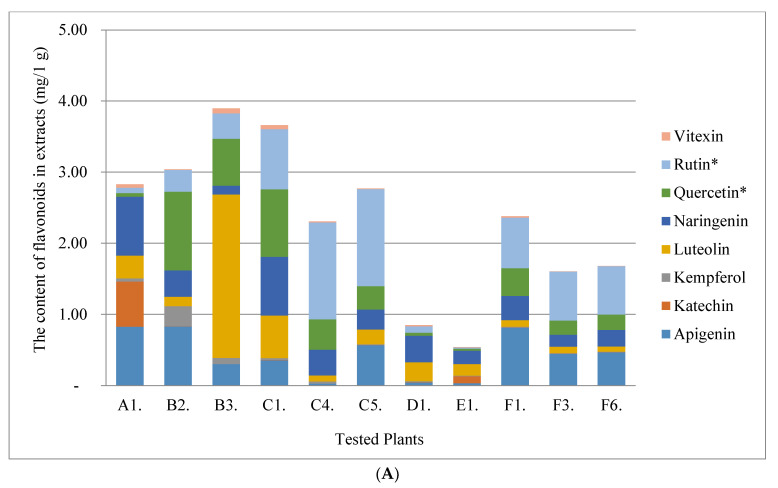

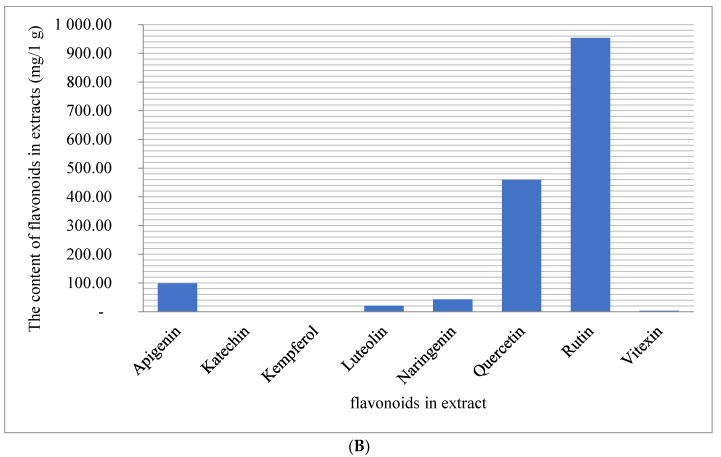
(**A**) The content of flavonoids in the plants tested. *—value for rutin and quercetin ×10. (**B**) the content of flavonoids in Elderberry fruit (*Sambucus nigra* L.) tested.

**Figure 2:**
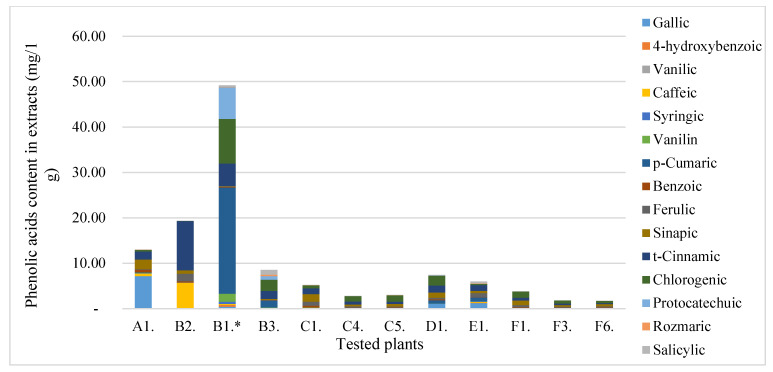
The content of phenolic acids in the plants tested. *—value for B1 × 10.

In the first stage, the content of total free phenolic acids (TPC) and total free flavonoids (TFC) (Table 1) of all plant’s raw materials was varied and ranged from 0.19 mg RUTE/1 g of extract to 38.50 mg RUTE/1 g of extract for TFC. In the case of TPC, the concentration range was similar in terms of the magnitude order of 1.35–60.96 (mg GAE/1 g of extract). In terms of the TPC and TFC content, the highest values were recorded for the beach rose fruit (TPC = 60.96 mg RUTE/1 g of extract; TFC = 38.30 mg RUTE/1 g of extract). The beach rose seeds and the elderberry flower were in second place in terms of content of the TPC and TFC. The lowest values were determined for elderberry leaves and sea buckthorn pomace. The antioxidant activity was correlated (r^2^ = 0.8625, r^2^ = 0.82437) with the TPC and TFC content, respectively (α = 0.05). Then, the bound flavonoids and phenolic acids content in the tested extracts were determined by the UPLC/PDA (ultra performance liquid chromatography (*UPLC*) with photodiode array detector (*PDA*)) method (Figure 1A,B and Figure 2). The results concerning flavonoids (Figure 1A,B) indicated that elderberry fruit extracts were characterized by several dozen times higher content than other plant materials. The dominant flavonoid was rutin. Elderberries had the highest content in the case of phenolic acids (Figure 2). The dominant phenolic acid was *p*-cumaric acid.

**Figure 3:**
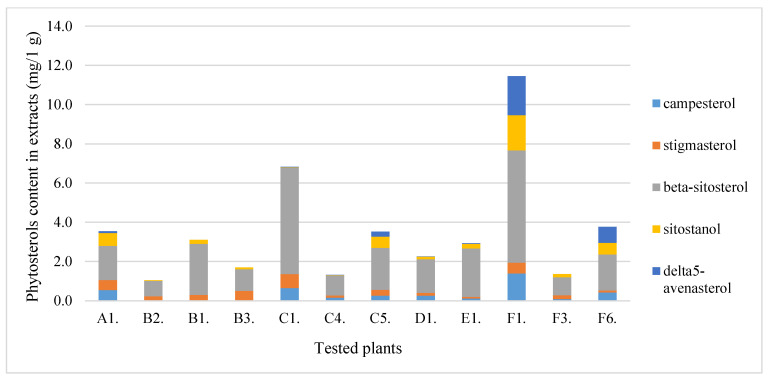
The content of sterols in the plants tested.

**Figure 4:**
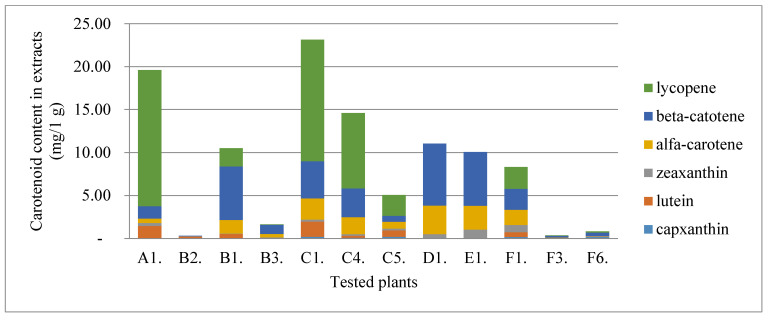
The content of carotenoids in the plants tested.

Sterols are compounds important not only in the diet of humans and animals. The tested plant materials are rich in phytosterols, and the extraction method allows for the effective extraction of these compounds. The content of the essential phytosterols was, therefore, determined (Figure 3). Sea buckthorn berries had the highest phytosterols content, followed by beach rose. The rest of the plant material contained similar amounts of sterols, and the dominant one was beta-sitosterol. Carotenoids were another group of hydrophobic compounds found in the extracts. The concentration of these compounds was, on average, about 10 mg/1 g of extract. The value of approx. 20 mg/1 g of the extract was recorded for bitter melon and beach rose (Figure 4).

**Figure 5:**
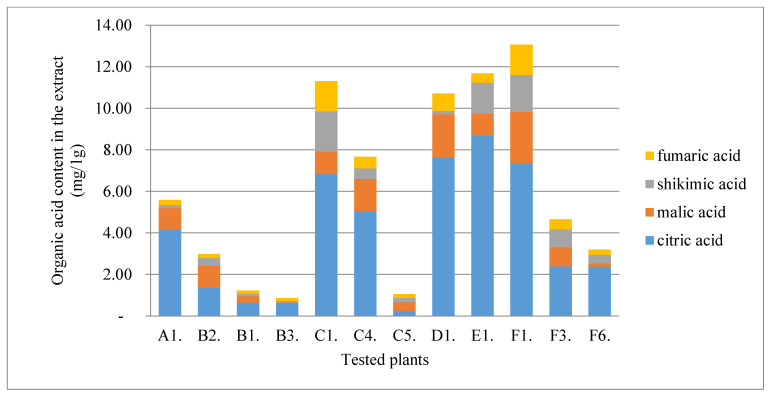
The content of organic acids in the tested plants.

Organic acids are the last group of essential compounds in plant raw materials. They determine the degree of fruit ripeness, taste, and the balance between alkalinity and acidity. Their activity as bioactive compounds is varied and not fully understood. The content of organic acids was about 1.5 mg/1 g of the extract to even 13 mg/1 g of the extract for sea buckthorn berries (Figure 5).

Together with the chemical analyses, microbiological studies on the antimicrobial activity of these extracts were carried out. Chemical studies showed the high antioxidant potential of all extracts as they contained a high concentration of bioactive compounds. In addition, seven strains of bacteria were tested, and MIGs were determined for each of them.

Based on the conducted research, it was found that polyphenol extracts from the tested raw materials concerning selected bacterial strains showed antibacterial activity in the tested concentration range (from 3.20% to 0.01%) (Table 2). The most sensitive to the extracts’ activity were the following bacteria: *M. luteus*, *P. mirabilis*, *P. fragii*, *S. enteritidis,* and *E. coli*. Earlier studies on the antimicrobial properties of elderberries showed that the extracts obtained with the same method described in this paper showed an inhibitory effect in the concentration range of 0.5% to 0.05% [18] on *M. luteus*, *P. mirabilis*, *P. fragii*, and *E. coli*. The studies conducted so far indicate that selected polyphenols and their esters are growth inhibitors of bacteria of the genera *Yersinia*, *Bacillus*, *Corynebacterium*, *Proteus*, *Staphylococcus*, *Enterococcus*, *Klebsiella*, *Micrococcus*, *Escherichia,* and *Pseudomonas*. Acids, such as gallic, vanillic, sinapic, and protocatechuic, inhibit the growth of Gram-positive and Gram-negative bacteria, e.g., *E. coli*, *Enterobacter cloacae* DG-6 and *Ps. Acidovorans* [19,20,21]. These acids are more effective against Gram-positive than Gram-negative bacteria. Bioactive compounds, such as caffeic, ferulic, and protocatechuic acids, also inhibit the growth of bacteria responsible for food poisonings, such as *Bacillus subtilis* and *Bacillus cereus* [22]. Kedzia and Hołderna-Kedzia [23] noted the inhibitory effect on the growth of *St. aureus*, *E. coli*, *K. pneumoniae*, *E. faecalis*, and *Ps. Aeruginosa* during their research. They argued that phenolic acids (p-coumaric, caffeic, and ferulic acids) showed antibacterial activity. Among other phenolic acids, gallic acid was also highly antibacterial (MIC for *S. aureus* was 150 μg/mL). On the other hand, the following acids: syrynic, gentisic, and *p*-hydroxybenzoic, had a weak effect on *S. aureus* (MIC > 1000 μg/mL) [23]. The latest literature on the subject also confirmed the antibacterial activity of six phenolic acids, and the antibacterial potential of the extracts against Gram-positive bacteria (*E. faecalis* and *L. monocytogenes*) was observed [24,25,26]. Camargo et al. [26] noted that phenolic acid extracts inhibited the growth of Gram-positive (*B. cereus*, *S. aureus*, *L. monocytogenes*, *Gb. Stearothermophilus*) and Gram-negative (*Ps. Aeruginosa*, *Ps. Fluorescens*, *S. enteritidis*, *S. typhimurium*, *E. coli*) bacteria [26]. The other researchers’ studies concerned the flavonoid extracts antibacterial activity against Gram-negative bacteria, i.e., *E. coli* and *Ps. Aeruginosa*. It was found that the activity level depended on their chemical structure [27]. Iwagawa et al. [28] noted that some quercetin derivatives inhibited the growth of *E. coli* [28]. On the other hand, Basile et al. [29] noted that apigenin, vitexin, and saponarin acted selectively against selected Gram-negative bacteria, i.e., *P. vulgaris*, *P. mirabilis*, *Ps. Aeruginosa*, *E. coli*, *K. pneumoniae*, and *E. cloacae* [29]. The minimum inhibitory concentration of apigenin was then determined. During their experiments, Waage and Hedin (1985) noted that quercetin-3-O-rhamnoside showed the highest activity against *Ps. Maltophilia* and *E. cloacae* [30]. Liu et al. [31] found that kaempferol glycosides are the most effective against Gram-positive bacteria. The MICs for the tested kaempferol and chloramphenicol glycosides against *B. cereus* were 16 µg/mL and 2 µg/mL, respectively [31]. In contrast, they were 32 μg/mL and 64 μg/mL, respectively, against Gram-positive *S. aureus* [32]. Based on their study of various methyl and acetyl flavonoids derivatives, Wang et al. [33] concluded that the presence of hydroxyl groups at the C-5 and C-7 positions was significant for antimicrobial activity. The presence of an additional methoxy group on C-7 or dihydroxy groups on C-3 and C-4 significantly reduced the antimicrobial activity [33].

The last stage of the research was the examined features statistical analysis of the extracts. The study aimed to define a model that would show the relationship between the analyzed chemicals, the extracts’ antioxidant activity, and MICs against the tested bacterial strains.

**Figure 6:**
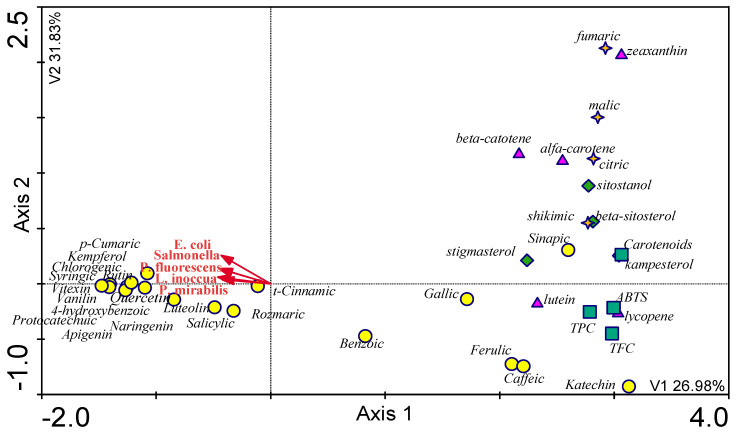
Canonical correlation analysis (CCA) (*n* = 38). Dependencies between groups of phenolic acids, organic acids, pigments, sugars, and their influence on the pathogenic and food spoilage microorganism’s development.

The model below (Figure 6, Table 3) clearly shows that higher concentration levels of test substances in plant organs may influence the pathogenic microorganism’s development and food spoilage. Indicators, such as ABTS, TPC, and TFC (total content of polyphenols and flavonoids), indicate that compounds from this group may reduce the activity of undesirable microorganisms. The compounds that can potentially affect the microorganism’s development include caffeic and ferulic acids, catechin, lutein, and lycopene.

The highest statistical significance for the model was found in the case of *Listeria innocua*, which may indicate the identified compounds may effectively reduce the bacterium activity.

**Figure 7:**
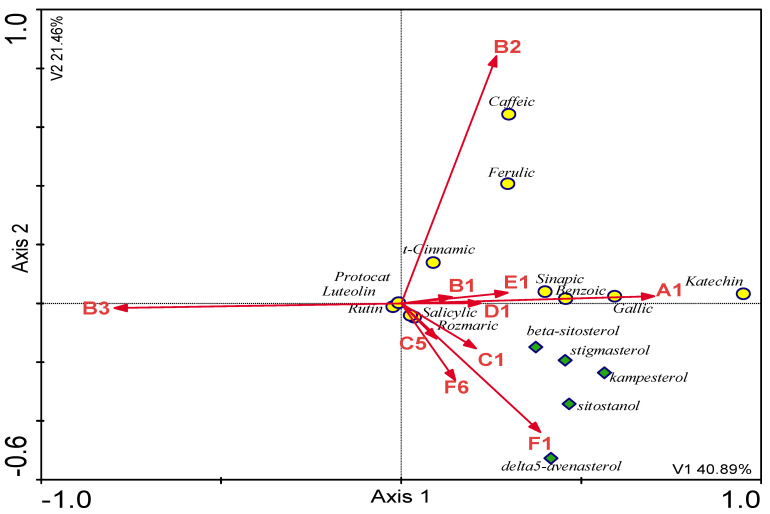
The CCA model. The tested compounds (polyphenols and sterols) distribution in plant material of the studied species.

By analyzing the models below, it can be concluded that the fruit turned out to be the richest in the tested substances out of all the analyzed plant parts. The bitter melon fruit contained the highest polyphenols concentrations (mainly catechin, which may influence the development of microorganisms). Moreover, both the bitter melon fruit and the sea buckthorn fruit had more significant amounts of sterols than the other tested plants’ fruit. The lowest polyphenols concentrations and sterols were recorded in elderberry leaves (Figure 7).

**Figure 8:**
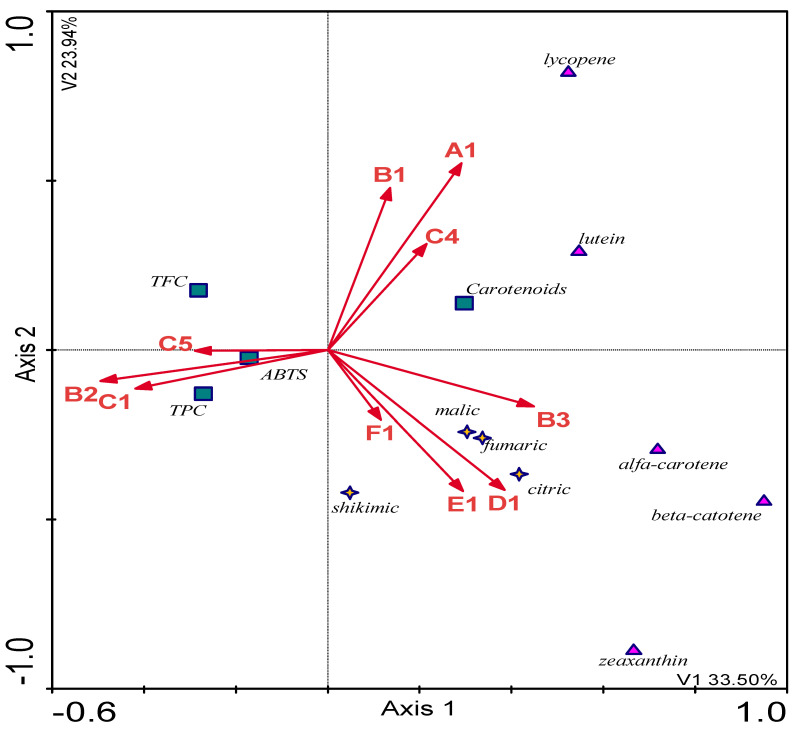
The CCA model. Distribution of the tested compounds (organic acids and carotenoids) in the plant material of the tested species.

In terms of general indicators of TFC, TPC, and ABTS^+^, the highest concentrations were recorded in fruits and seeds of beach rose and elderberry flowers. In addition, higher concentrations of organic acids were found in the rowan, guelder rose, and sea buckthorn fruits. In contrast, the highest content of lycopene and lutein was recorded for the bitter melon and elderberry fruits (Figure 8).

In the case of bitter melon, the conducted studies showed the antimicrobial effect of *Momordica charantia*. The experiment performed by researchers proved that in this species of fruits, ethanol extracts inhibit the growth of many bacteria: *Bacillus cereus* RSKK 863, *Escherichia coli* O157: H7, *Shigella sonnei* Mu: 57, *Micrococcus luteus* NRRL B-4375, *Yersinia enterocolitica* NCTC 11175, *Escherichia coli* ATCC 11229, *Pseudomonas aeruginosa* ATCC 27853, *Staphylococcus aureus* TCC 25923, *Escherichia coli* ATCC 35218, *Salmonella enteritidis* ATCC 13076, *Listeria monocytogenes* ATCC 7644, and the yeast *Candida albicans* ATCC 10231. The unripe fruit extract was characterized by a higher activity against the tested microorganisms than the ripe fruit extract. The same study found antimicrobial activity against fish pathogens, such as *Lactococcus garvieae*, *Yersinia ruckeri*, *Vibrio anguillarum* M1, *Vibrio anguillarum* A4, and *Vibrio alginolyticus* [19,27]. An experiment by Kilcar et al. [34] showed that both bitter melon fruit ethanolic extracts and seeds and seed oil extracts have antibacterial activity against *Escherichia coli*, *Pseudomonas aeruginosa,* and *Salmonella typhi* and antifungal activity against *Aspergillus niger* and *Candida albicans*. Mahmood et al. [35] confirmed the various extracts’ antimicrobial activity (water, ethanol, ether, and n-hexane) from bitter melon seeds against all tested microorganisms: *Escherichia coli*, *Klebsiella pneumoniae*, *Lactobacillus bulgaricus*, *Micrococcus luteus*, *Pseudomonas aeruginosa*, *Pasteurelotelaus vulocida*, *Pasteurelotelaus vulocida aureus*, *Staphylococcus epidermidis*, and *Salmonella typhi*. The effect depended on the extract type used. The antioxidant activity of *Momordica charantia* is characterized by a high antioxidant potential, which results from the presence of the polyphenolic compound contained in plant various parts, among others causes. The experiment carried out by Kubol and Siriamornpun [36] proved the bitter melon leaves, stems, and unripe and ripe fruits water extracts are characterized by the ability to scavenge the synthetic DPPH (2,2-difenylo-1-pikrylohydrazyl) radical. The highest antioxidant activity was found for leaves (89.3%) and the lowest for ripe fruit (55.5%). On the other hand, the FRAP (ferric reducing antioxidant power) test conducted by Ozusaglam and Karakoca [37] proved the ripe fruit and seeds ethanol extracts had the highest reducing force. Scientists obtained similar results in the CUPRAC (cupric reducing antioxidant capacity) method, based on the reduction of the neocuproine complex with Cu(II) ions. In studies carried out on rats, it was noted that individuals that received intraperitoneal bitter melon extract showed a statistically significantly higher concentration of antioxidant enzymes, such as catalase (CAT), superoxide dismutase (SOD), glutathione S-transferase (GST), and glutathione peroxidase (GPx) in liver, kidney, and lung tissues compared to control animals. As shown by Wu and Ng [38], administering a bitter melon aqueous and ethanolic extract to rats inhibited lipid peroxidation induced by iron chloride and ascorbic acid, both in the liver, brain, and blood plasma.

*Rosa rugosa* Thunb. belongs to the most valuable representatives of the rose family (*Rosaceae*) found in Poland in nutritional value. In the wild, *Rosa rugosa* Thunb. shrubs occur in various areas, showing resistance to harsh environmental conditions (poor soils, limited water access). Growing beach roses is not possible only in acidic soils and wetlands. *Rosa* L. genus fruits, which include the species *Rosa canina* L. and *Rosa rugosa* Thunb., are characterized by a high content of various biologically active compounds [10]. They contain large amounts of vitamin C, as well as tocopherols and carotenoids. The fruits of these species are also a significant source of such macronutrients as P, K, Ca, and Mg. *Rosa canina* L. fruits are also a valuable source of simple sugars (fructose, glucose, and trehalose) and polysaccharides—pectins. Other polar compounds found in the beach rose fruit include phenolic acids (gallic and genistic), showing antifungal and antiviral properties. Due to their rich composition, beach roses are used in the food, pharmaceutical, cosmetic, herbal medicine, and aromatherapy industries. They are used in liver and gallbladder diseases, kidney diseases, colds, hyperacidity, and peptic ulcer disease in herbal medicine.

The antibacterial effect of elderberry flower and fruit extracts has been confirmed in numerous laboratory tests. It turned out that standardized fruit extract at a 10% concentration in liquid culture inhibits the growth of streptococci (including *Streptococcus pyogenes*) responsible for upper respiratory tract infections and strains of *Moraxella catharalis* [39]. In the study by Herst et al. [40], the significant activity of elderberry leaf and fruit extracts against numerous nosocomial pathogens was found, including against methicillin-resistant strains of *Staphylococcus aureus* MRSA, as well as Gram-negative bacteria (*Salmonella poona*, *Pseudomonas aeruginosa*). Moreover, Sadeghi-Kiakhani et al. [41] observed the growth inhibition of *Bacillus subtilis*, *Staphylococcus aureus*, *Salmonella typhi*, *Pseudomonas aeruginosa*, *Escherichia coli,* and *Candida albicans* yeast strains by using the methanolic elderberry extract [41]. The tannins (gallic, hydroxycinnamic, and coffee acids derivatives) and triterpenes (alpha and beta amyrin, oleanolic acid) are most likely responsible for the antibacterial activity of *Sambucus nigra* L. extracts [40].

Liepiņa et al. [42] assessed the antimicrobial effect of rowan fruit extracts. The extracts showed activity against *Bacillus cereus*, *Staphylococcus aureus,* and *Pseudomonas aeruginosa*, but no action against *Escherichia coli* was found. The rowan antimicrobial activity has previously been attributed to sorbic acid, which has an inhibitory effect, especially on yeasts and molds [43]. Chlorogenic acids (3- and 5-caffeoylquinic acid) account for approximately 80% of all phenols found in wild rowan trees. For comparison, cultivated rowan trees contain fewer caffeoylquinic acids and more anthocyanins [44].

In the case of guelder rose fruit, the research by Sagdic et al. [42] showed the 15% concentration extracts inhibited the growth of *A. hydrophila*, *B. cereus*, *E. aerogenes*, *E. coli*, *K. pneumoniae*, *P. vulgaris*, *P. aeruginosa*, *S. typhimurium*, *S. aureus,* and *Y. enterocolitica*. On the other hand, the 5% extracts concentration showed inhibitory activity only against *A. hydrophila* and *S. aureus*. Similar antimicrobial activity of guelder rose fruit extracts was obtained by Sagdic [45], Ozcan et al. [46], and Baydar et al. [47].

Research by Yue et al. [48] showed that sea-buckthorn fruit extracts (essential oils) inhibited the growth of five bacteria species: *Bacillus subtilis*, *Bacillus cereus*, *Bacillus coagulans*, *Staphylococcus aureus*, and *Escherichia coli*. In this study, various elements of sea buckthorn berries were examined: pomace, seeds, and leaves. The extracts from the different parts showed an almost equal inhibitory effect on *S. aureus*. The pomace extract was the most effective, followed by the seeds and leaves for the *Bacillus subtilis*, *Bacillus cereus*, and *Bacillus coagulans* bacteria. The seed extract shows a better inhibitory effect than the leaf or pomace extract for *E. coli*.

Literature analysis showed that many scientists used mainly ethanol or ethanol–water extracts in their research. The method developed by our team allows, thanks to double hydrolysis, the release of all antioxidant compounds. Moreover, it will enable the use of water to digest dry extracts and eliminate the effect of ethanol as an antimicrobial substance. Ethanol extracts obtained from fresh, dried, or frozen plant materials show different pH and antibacterial properties. Freezing the fruit leads to a reduction in their antibacterial effect. Vacuum freeze-drying is the best industrial method of preparing plant materials [49], but it is expensive. The results of the research indicate that pH is not the factor determining the extracts’ antimicrobial activity. At the same time, it is true that organic acids, e.g., ascorbic acid, have a strong antimicrobial effect [50]. An essential role in providing plants with antimicrobial properties is played by individual substances and the entire complex of biologically active compounds. For example, ascorbic acid has a synergistic antimicrobial effect in combination with a low pH [51]. The use of extraction immediately after harvesting will reduce the loss of bioactive compounds and lower the antioxidant and antimicrobial activity. Anhydrous extracts can be stored under refrigerated conditions for a long time without losing their properties.

## 3. Materials and Methods

### 3.1. Tested Material

The tested material was various plant parts (leaves, flowers, fruits) collected in the period between 2021–2022:

1. Rowan fruits (*Sorbus aucuparia* L.) were collected from natural habitats in October 2022.
2. Guelder rose fruits (*Viburnum opulus* L.) were collected from natural habitats in October 2022.
3. Sea buckthorn fruits (*Hippophaë rhamnoides* L.) were collected from natural habitats in September 2022.
4. Bitter melon fruits (*Momordica charantia* L.) were collected from natural habitats in September 2021.
5. Elderberry (*Sambucus nigra* L.) flowers were collected from natural habitats in June 2022.
6. Beach rose (*Rosa rugosa Thunb*.) leaves were collected from natural habitats in July 2022.

The material for the study was collected from natural habitats in the Forest Arboretum in Zielonka—Murowana Goślina (17°01′ E, 52°34′ N).

The bitter melon fruits were washed, dried on tissue paper, cut into 5 mm thick slices, and freeze-dried. Then, they were stored in tightly closed glass vessels in a freezer (−20 °C) in the dark until chemical analyses were performed.

Plant materials, such as elderberry flowers and beach rose leaves, were dried in a dryer at a temperature of 40 °C (for several hours) after removing the moldy fragments. Next, they were mechanically crushed, transferred to glass jars, and stored in the freezer without access to light (−20 °C) until chemical analyses were performed.

After removing the unripe or moldy parts, the rowan, guelder rose, and sea buckthorn fruits were subjected to preliminary mechanical treatment to obtain the appropriate fractions. The plant material was freeze-dried. It was stored in a freezer (−20 °C) in the dark until chemical analyses were performed.

### 3.2. Extraction Procedure

An amount of 10 g of dehydrated plant material was placed in a round-bottom 750 mL flask. The flask was placed under a reflux condenser. Alkaline hydrolysis was conducted first, which was followed by acid hydrolysis. An amount of 50 mL of distilled water and 200 mL of a two-molar aqueous sodium hydroxide solution were added to test tubes to perform the alkaline hydrolysis. Next, the contents were heated in a heating mantle at 95 °C for 30 min. After cooling (about 20 min), the samples were neutralized with 100 mL of a six-molar aqueous solution of hydrochloric acid (pH = 2). Next, the samples were cooled in water with ice. Phenolic compounds were extracted from the inorganic phase with diethyl ether (2 × 100 mL). The obtained ether extracts were continuously transferred into round-bottom 250 mL distillation flasks and evaporated in a rotary evaporator.

Next, acid hydrolysis was conducted. An amount of 150 mL of a six-molar aqueous solution of hydrochloric acid was added to the aqueous phase and heated again at 95 °C for 30 min. After cooling in water with ice, the samples were extracted with diethyl ether (2 × 100 mL). The obtained ether extracts were continuously transferred into round-bottom 250 mL distillation flasks and evaporated in a rotary evaporator. The extract was quantitatively transferred from the distillation flasks into 8 mL vials while washing the flasks with 2 × 4 mL diethyl ether. Next, the extracts were dried under a stream of nitrogen at room temperature. The dry extracts were weighed and stored at −80 °C until further analysis (Table 1). Before the following stages of the analysis, the extracts were thawed under refrigerated conditions for 12 h and then digested in a selected volume of distilled water of MiliQ purity to obtain the chosen concentration. The elderberry extracts were digested to a volume of 25 mL in a volumetric flask.

The extracts were obtained by an innovative method as double hydrolysis of bioactive compounds (acid and alkaline) was used. After each hydrolysis, the bioactive compounds that were released from the glycosidic bonds were extracted with diethyl ether. This allowed for the precise determination of the qualitative and quantitative profile of bioactive compounds in the tested samples.

After evaporation, the dry extracts were dissolved in deionized water and those derived from alkaline and acid hydrolysis were combined and then used in microbiological studies.

The extracts were tested for the minimum inhibitory concentration (MIC) against seven bacterial strains and analyzed chemically to determine the content of phenolic compounds, pigments, and antioxidative properties in a test with the ABTS^+^ radical.

### 3.3. Total Phenolic Acids and Total Flavonoids

The obtained extracts of bioactive compounds were analyzed for the content of total phenolic compounds, total phenolic acids, and total flavonoids using a modified spectrophotometric method, which was adapted to liquid chromatography conditions. The total polyphenol content was tested using the Folin–Ciocalteu reagent. A sample (40 µL) of the dissolved extract mixture in 1 mL of Me/H_2_O (1:1, *v*/*v*) was added to 3 mL of distilled water and 200 µL of Folin–Ciocalteu reagent and mixed. The mixture was shaken and allowed to stand for 6 min, then 600 µL of a sodium carbonate solution was added and shaken to mix. The solutions were left at 20 °C for 2 h. The samples were then filtered through a 0.45 µm filter. Total bioactive compounds were analyzed using an Acquity UPLC liquid chromatography (Waters, Milford, MA, USA) with a Waters Acquity PDA detector (Waters, Milford, MA, USA) on an Acquity UPLC^®^ BEH C18 column (100 mm × 2.1 mm, particle size 1.7 μm) (Waters, Dublin, Ireland) at a 320 nm wavelength for total phenolic acids, 765 nm wavelength for total phenolic compounds, and 624 nm wavelength for total flavonoids, relative to the blind test. The sample injection volume was 10 µL, and the flow rate was 0.4 mL/min. The isocratic elution was performed using a mobile phase, which was a mixture of A: 0.1% formic acid solution in acetonitrile and B: 0.1% aqueous formic acid solution [52]. The peak areas were summed to determine the total polyphenol content. The total phenolic acid content of the extracts (three replicates per treatment) was expressed as mg gallic acid equivalents per gram (mg GAE/g) by comparison with the caffeic acid calibration curve. The range of the calibration curve was 10–1000 mg/L (r^2^ = 0.9982). The total flavonoid extracts content (three replicates per treatment) was expressed as mg rutin equivalents per gram (mg RUTE/g) by comparison with the rutin calibration curve. The range of the calibration curve was 10–1000 mg/L (r^2^ = 0.9871). Each commercial compound standards was obtained from Sigma (St. Louis, MO, USA). The obtained results were converted into 1 g of extract [52].

### 3.4. ABTS^+^ Antioxidant Activity

The ABTS^+^ cation radicals method was used to determine the antioxidant activity of the extracts. A 2 mm ABTS^+^ stock solution containing 3.5 mM potassium sulfate (VI) was prepared by diluting the stock solution eight times in methanol and overnight incubation at room temperature in the dark to allow radical stabilization. An Acquity UPLC liquid chromatography system (Waters, Milford, MA, USA) with a Waters Acquity PDA detector (Waters, Milford, MA, USA) and an Acquity UPLC^®^ BEH C18 column (150 mm × 2.1 mm, particle size 1.9 μm) (Waters, Dublin, Ireland) were used. The gradient started with A: 0.1% aqueous formic acid solution in acetonitrile and B: 0.1% aqueous formic acid (10:90, %, *v/v* to 40:60, %, *v/v*) for 15 min. The chromatogram was recorded at a 280 nm wavelength. The injection volume of the sample was 10 µL, and the flow rate was 0.4 mL/min. After running through the column, the UPLC elution returned to baseline. The sample was then re-injected with ABTS^+^. This time the chromatogram was recorded at a 734 nm wavelength. The antioxidant activity was calculated from the difference in peak areas of both chromatograms and compared with the standard curve for TROLOX. Each commercial compound standards was obtained from Sigma (St. Louis, MO, USA). The obtained results were converted into 1 g of extract [53,54].

### 3.5. Determination of LMWOA (Low Molecular Weight Organic Acid)

LMWOA’s analysis was performed using a class H Waters Acquity UPLC system. Separation was achieved on an Acquity UPLC BEH C18 column (150 mm × 2.1 mm, 1.7 µm, Waters, Milford, MA, USA), thermostated at 35 °C. The gradient elution was performed with water and acetonitrile (both containing 0.1% formic acid, pH = 2) at a 0.4 mL/min flow rate. Detection was performed on a Waters Photodiode Array Detector (Waters Corporation, Milford, MA, USA) at λ = 280 nm as the preferred wavelength for oxalic, malonic, citric, malic, quinic, and shikimic acids. Compounds were identified by comparing the analyzed peaks retention time with the standards or by adding a specific standard to the analyzed samples and reanalyzing. The retrieved LMWOAs were quantified by comparing the area of their peaks recorded at 280 nm wavelength with calibration curves obtained from each commercial compound standards was obtained from Sigma (St. Louis, MO, USA). The obtained results were converted into 1 g of extract [55].

### 3.6. Carotenoids Determination

Carotenoid isolation and quantification were performed in grain samples by saponification in Acquity UPLC (Waters, Milford, MA, USA). Carotenoid extracts were obtained from fragmented samples (0.4 mg) and triturated with acetone and petroleum ether (1:1). Then, after separating the plant tissue, acetone and the hydrophilic fraction were removed from the extract by washing with water. As a result, an ether extract with a mixture of carotenoid pigments was obtained. The prepared extract was concentrated in a vacuum evaporator at 35 °C until an oily residue was obtained, then it was digested in 2 mL of methanol (Merck) and subjected to chromatographic analysis. Total carotenoid content was determined using Acquity UPLC (Waters, Milford, MA, USA) with a Waters Acquity PDA detector (Waters, Milford, MA, USA). The chromatographic separation was performed on an Acquity UPLC^®^ BEH C18 column (100 mm × 2.1 mm, particle size 1.7 µm) (Waters, Dublin, Ireland). Elution was performed with solvent A—methanol, B—water, and methyl tertiary-butyl ether (MTBE). A gradient was applied at a 0.4 mL/min flow. The column and samples were thermostated. The column temperature was 30 °C, and the test temperature was 10 °C. The solutions were degassed in a Waters apparatus during the analysis. The injection volume was 10 µL. The recording was carried out at the wavelength λ = 445 nm. Each commercial compound standards was obtained from Sigma (St. Louis, MO, USA). The obtained results were converted into 1 g of extract [56].

### 3.7. Phenolic Compounds Determination

Phenolic compounds in samples were analyzed after alkaline and acidic hydrolysis (53, 54). Analysis was performed using an Acquity H class UPLC system equipped with a Waters Acquity PDA detector (Waters, Milford, MA, USA). Chromatographic separation was performed on an Acquity UPLC^®^ BEH C18 column (100 mm × 2.1 mm, particle size 1.7 μm) (Waters, Dublin, Ireland). The elution was calculated using the following mobile phase composition: A: acetonitrile with 0.1% formic acid and B: 1% aqueous formic acid mixture (pH = 2). Concentrations of phenolic compounds were determined using an internal standard at wavelengths λ = 320 nm and 280 nm. Compounds were identified based on a comparison of retention time of the analyzed peak with the retention time of the standard and by adding a specific amount of the standard to the analyzed samples and repeating the analysis. The detection level is 1 μg/g. Retention times and recovery rates for the analyzed phenolic compounds are in Table 4. Each commercial compound standards was obtained from Sigma (St. Louis, MO, USA). The obtained results were converted into 1 g of extract [54,57,58].

### 3.8. Sterol Analysis

Samples weighing about 10 g were taken for analysis. After grinding with a laboratory mill (WŻ-1), 0.1 g samples were taken for further research. The samples were placed in tightly sealed 17 mL culture tubes in which sterol extraction was performed with simultaneous saponification. These processes took place under the influence of microwave radiation. For this purpose, methanol (2 mL) and a 2 M aqueous sodium hydroxide solution (0.5 mL) were added to the tubes. Tightly sealed culture tubes were placed in plastic bottles for safety, which, in turn, were placed in a microwave oven (Whirlpool model AVM 401/1/WH, 2450 MHz, 900 W). The tests were exposed to the microwave radiation of 350 W power in two sessions of 20 s each. After cooling (about 15 min), the samples were neutralized with a 1 M aqueous solution of hydrochloric acid. Then, after adding 2 cm^3^ of methanol, the sterols were extracted with pentane (3 × 4 mL). The combined pentane extracts were collected in an 8 mL vial and evaporated to dryness under a nitrogen stream. Before the chromatographic analysis, the samples were dissolved in 1 mL of methanol, centrifuged, and the supernatant was filtered using syringe filters (0.22 µm). Qualitative and quantitative analysis of sterols was performed using ultra-efficient liquid chromatography (Waters Acquity UPLC, Milford, MA, USA) with a PDA detector (Waters Acquity UPLC, Milford, MA, USA). The chromatographic separation was carried out on an Acquity UPLC^®^ BEH C18 column (100 mm × 2.1 mm, particle size 1.7 μm), and elution was carried out in an isocratic manner using the following mobile phase composition: A acetonitrile 10%, B methanol 85%, and C water 5%, with a 0.5 mL/min flow. The sterols concentration measurement (campesterol → stigmasterol → beta-sitosterol → sitostanol → delta5-avenasterol) was carried out using the standard external method at the wavelength λ = 210 nm. Identification of sterols was carried out by comparing the retention time of the tested peak with the retention time of the original standard and by adding a specific amount of the standard to the test sample and reanalyzing it. The detection limit was 0.1 mg/kg. Each commercial compound standards was obtained from Sigma (St. Louis, MO, USA). The obtained results were converted into 1 g of extract [59].

### 3.9. MIC Measurement

The minimum inhibitory concentrations (MICs) of the raw material extracts inhibiting the growth of *Escherichia coli* (PCM 2793), *Salmonella enteritidis* (PCM 2548), *Proteus mirabilis* (PCM 1361), *Pseudomonas fluorescens* (PCM 2123), *Pseudomonas fragii* (PCM 1856), *Listeria innocua* (DSM 20649), and *Micrococcus luteus* (PCM 525) were measured with a Bioscreen C automated growth reader. Bacterial inoculants and a series of diluted hydrated extracts were placed on a plate and incubated for 72 h under adequate conditions for individual groups of bacteria. The apparatus measured the optical density (OD) in each cuvette. The first concentration without turbidity was considered the MIC value, i.e., the minimum concentration at which the microorganisms used in the study were inactivated during 72 h incubation.

### 3.10. Statistical Analysis

Statistical analyses and models were based on discriminant analysis. The analyses showed which of the variables—pigments, sugars, organic acids, or polyphenols, may affect the activity of pathogenic and food spoilage microorganisms. The model was constructed using canonical variate analysis (CVA)—the canonical variation of Fisher’s linear discriminant analysis (LDA) (Šmilauer and Lepš 2003). The borderline significance level was determined with the Monte Carlo permutation test (number of permutations: 9.999). The Canoco 5 for Windows package and the Microsoft Excel spreadsheet (accessed 3 October 2019) were used for all comparisons, calculations, and graphic elements. In addition, the following tools from Canoco for Windows were used: Canoco for Windows 4.5, CanoDraw for Windows, and WCanoImp (Microcomputer Power, New York, USA).

The following identifiers were made for the tested plants (Table 5).

## 4. Conclusions

Plant extracts are a source of valuable ingredients that can be used in many industries. The paper presents the research on the content of selected bioactive compounds in extracts obtained from various plant materials. Raw materials rich in polyphenols, which have documented use in traditional medicine, were selected. The tested plants and raw materials were: bitter melon (fruits), elderberry (flowers, fruits, leaves), wild rose (fruits, pulp, seeds), mountain ash (fruits), viburnum (fruits), sea buckthorn (fruits, leaves, pomace). The aim of these studies was to identify potential raw materials that may be an alternative source of bioactive compounds with antibacterial activity. The following bacteria were the most sensitive to the action of the tested extracts: M. luteus, P. mirabilis, P. fragii, S. enteritidis, and E. coli. The tested plant materials can be used in various industries as a source of bioactive antibacterial compounds. However, further research is needed to identify the individual biologically active components of these raw materials.

## Figures and Tables

**Table 1:** The total phenolic content (TPC (mg GAE/1 g extract)), total flavonoid content (TFC (mg RUTE/1 g extract)), and ABTS^+^ ((2,2′-azino-bis(3-ethylbenzothiazoline-6-sulfonic acid (µmolTROLOX/1 g extract s.m.)) in the plants tested.

Nr.	Plants	TPC (mg GAE/1 g Extract)	ABTS (µmoLTROLOX/1 g Extract s.m.)	TFC (mg RUTE/1 g Extract)
A1.	Bitter melon fruits (*Momordica charantia* L.)	11.61	11.81	3.74
B2.	Elderberry flower (*Sambucus nigra* L.)	34.98	50.28	21.52
B1.	Elderberry fruit (*Sambucus nigra* L.)	9.64	33.15	18.09
B3.	Elderberry leaves (*Sambucus nigra* L.)	1.35	1.66	0.21
C1.	Beach rose fruit (*Rosa rugosa Thunb.*)	60.96	47.10	38.30
C4.	Beach rose pulp (*Rosa rugosa Thunb.*)	2.01	9.39	17.95
C5.	Beach rose pips (*Rosa rugosa Thunb.*)	31.06	23.54	15.09
D1.	Rowan fruits (*Sorbus aucuparia* L.)	7.59	10.18	0.23
E1.	Guelder rose fruits (*Viburnum opulus* L.)	12.03	14.07	0.42
F1.	Sea-buckthorn fruits (*Hippophaë rhamnoides* L.)	15.59	16.22	4.18
F3.	Sea-buckthorn leaves dried in the sun (*Hippophaë rhamnoides* L.)	5.03	5.34	2.51
F6.	Sea-buckthorn pomace after juice-thermal treatment (*Hippophaë rhamnoides* L.)	1.77	3.61	1.05
	min	1.31	1.6	0.19
	max	61.04	50.86	38.5
	mean	16.3	18.86	10.27
	SD	17.29	15.95	11.64

Total phenolic content (TPC (mg GAE/1 g extract)), Total flavonoid content (TFC (mg RUTE/1 g extract)), ABTS^+•^ ((2,2′-azino-bis(3-ethylbenzothiazoline-6-sulfonic acid (µmoLTROLOX/1 g extract s.m.)). GAE gallic acid, RUTE routine, TROLOX 6-hydroxy-2,5,7,8-tetramethylchroman-2-carboxylate acid, s.m. dry mass.

**Table 2:** The minimum inhibitory concentrations (MICs) [%] of the plants’ extracts.

		Tested Bacteria						
		***Escherichia coli***	***Salmonella enteritidis***	***Proteus mirabilis***	***Pseudomonas fluorescens***	***Pesudomonas fragii***	***Listeria innocua***	***Micrococcus luteus***
		**MIC**	**MIC**	**MIC**	**MIC**	**MIC**	**MIC**	**MIC**
*Momordica charantia* L.	SD	0.02	0.02	0.02	0.02	0.02	0.01	0.00
	Min	0.03	0.05	0.1	0.05	0.05	0.05	0.05
	Max	0.08	0.025	0.075	0.075	0.025	0.05	0.05
	Mean Value	0.05	0.05	0.10	0.05	0.05	0.06	0.05
*Sambucus nigra* L. (*Sambuci flos*)	SD	0.04	0.05	0.01	0.01	0.03	0.02	0.00
	Min	0.13	0.1	0.175	0.15	0.05	0.11	0.05
	Max	0.20	0.2	0.2	0.175	0.1	0.15	0.05
	Mean Value	0.16	0.16	0.18	0.15	0.08	0.13	0.05
*Sambucus nigra* L. (*Sambuci fructus*)	SD	0.01	0.06	0.03	0.05	-	0.03	0.00
	Min	0.10	0.1	0.1	0.1	0.15	0.1	0.05
	Max	0.13	0.2	0.15	0.15	0.152	0.15	0.05
	Mean Value	0.11	0.13	0.13	0.15	0.15	0.12	0.05
*Sambucus nigra* L. (*Sambuci folium*)	SD	0.03	0.03	0.01	0.03	0.02	0.03	0.03
	Min	2.35	2.65	2.4	1.7	1.7	2.55	1.4
	Max	2.40	2.7	2.425	1.75	1.73	2.6	1.45
	Mean Value	2.38	2.67	2.43	1.73	1.72	2.57	1.43
*Rosa canina* L. (*Fructus Rosae*)	SD	0.03	0.01	0.01	0	0.01	0.03	0.01
	Min	0.10	0.1	0.15	0.05	0.05	0.1	0.05
	Max	0.15	0.125	0.175	0.051	0.075	0.15	0.075
	Mean Value	0.12	0.12	0.15	0.05	0.07	0.12	0.06
*Rosa canina* L. (*Rosa impetus*)	SD	0.05	0.05	0.03	0.05	0.05	0.03	0.00
	Min	0.60	0.8	0.8	0.55	0.4	0.075	0.05
	Max	0.70	0.9	0.85	0.6	0.5	0.125	0.05
	Mean Value	0.65	0.85	0.82	0.55	0.45	0.10	0.05
*Rosa canina* L. (*Rosa ravageur*)	SD	0.01	0.01	0.01	0.01	0.01	0.01	0.00
	Min	0.05	0.075	0.1	0.05	0.05	0.05	0.05
	Max	0.08	0.1	0.125	0.075	0.075	0.075	0.05
	Mean Value	0.06	0.09	0.11	0.06	0.06	0.07	0.05
*Sorbus aucuparia* L. (*Fructus*)	SD	0.03	0.02	0.03	0.05	0.03	0.03	0.01
	Min	0.50	0.25	0.3	0.2	0.225	0.25	0.1
	Max	0.55	0.3	0.35	0.3	0.275	0.3	0.075
	Mean Value	0.53	0.28	0.32	0.26	0.25	0.28	0.08
*Viburnum opulus* L. (*Fructus*)	SD	0.01	0.01	0.02	0.03	0.03	0.01	0.01
	Min	0.20	0.3	0.3	0.15	0.15	0.2	0.1
	Max	0.18	0.31	0.25	0.175	0.15	0.225	0.075
	Mean Value	0.20	0.3	0.275	0.2	0.2	0.2	0.075
*Hippophae rhamnoides* L. (*Fructus*)	SD	0.03	0.10	0.03	0.03	0.04	0.03	0.03
	Min	0.50	1.75	1.2	0.85	0.8	1	0.45
	Max	0.50	1.8	1.25	0.9	0.725	1	0.4
	Mean Value	0.52	1.72	1.23	0.87	0.77	0.98	0.42
*Hippophae rhamnoides* L. (*Folium*)	SD	0.03	0.05	0.03	0.03	0.03	0.05	0.06
	Min	2.95	3.1	2.9	2.25	2.2	2.8	2
	Max	3.00	3.2	2.95	2.3	2.25	2.9	2.1
	Mean Value	2.97	3.15	2.92	2.27	2.23	2.85	2.07
*Hippophae rhamnoides* L. (pomace)	SD	1.47	1.55	1.44	1.12	1.10	1.40	1.01
	Min	2.90	3.1	2.9	2.2	2.25	2.85	2.15
	Max	3.00	3.2	2.95	2.25	2.3	2.85	2.25
	Mean Value	2.95	3.15	2.90	2.23	2.27	2.83	2.18

**Table 3:** Statistical parameters for the CCA analysis (Figure 6).

Variable	*p*-Value	*F*-Value	[%] Expl.
*Listeria innocua*	46.848	0.001	41.74
*Escherichia coli*	4.243	0.002	12.41
*Proteus mirabilis*	3.315	0.020	5.56
*Pseudomonas fluorescens*	3.487	0.011	6.53
*Salmonella enteritidis*	4.313	0.014	10.41

**Table 4:** Retention times and recovery rates for the analyzed phenolic compounds.

Analyzed Phenolic Compounds	Retention Times [min]	Recovery Rates [%]
aempferol	6.11	86 ± 5.3
gallic acid	8.85	92 ± 4.4
vanillic	9.71	79 ± 8.5
luteolin	11.89	96 ± 2.7
protocatechuic acid	12.23	90 ± 4.8
vanillin acid	14.19	88 ± 5.1
apigenin	16.43	93 ± 3.8
catechin	18.09	89 ± 5.7
4-hydroxybenzoic acid	19.46	96 ± 3.78
chlorogenic acid	21.56	92 ± 2.8
caffeic acid	26.19	86 ± 6.7
syringic acid	28.05	94 ± 3.9
naringenin	31.22	88 ± 4.8
vitexin	35.41	95 ± 3.8
rutin	38.11	93 ± 4.9
quercetin	39.58	97 ± 1.9
p-coumaric acid	40.20	89 ± 3.6
ferulic acid	46.20	91 ± 4.9
sinapic acid	48.00	94 ± 5.1
t-cinnamic acid	52.40	97 ± 2.9

**Table 5:** Designation of the tested species used in the models.

Species	Designation	Plant Material	Designation
Bitter melon fruits (*Momordica charantia* L.)	A	Fruit	1
Elderberry (*Sambucus nigra* L.)	B	Flower	2
Beach rose (*Rosa rugosa Thunb.*)	C	Leaves	3
Rowan fruits (*Sorbus aucuparia* L.)	D	Pulp	4
Guelder rose fruits (*Viburnum opulus* L.)	E	Pips	5
Sea buckthorn fruits (*Hippophaë rhamnoides* L.)	F	Pomace	6

## Data Availability

Not applicable.

## Sample Availability

Samples of the compounds are available from the authors.
